# Chemical ordering in substituted fluorite oxides: a computational investigation of Ho_2_Zr_2_O_7_ and RE_2_Th_2_O_7_ (RE=Ho, Y, Gd, Nd, La)

**DOI:** 10.1038/srep38772

**Published:** 2016-12-12

**Authors:** Jonathan M. Solomon, Jacob Shamblin, Maik Lang, Alexandra Navrotsky, Mark Asta

**Affiliations:** 1 Department of Materials Science and Engineering, University of California Berkeley, Berkeley, CA 94720, United States; 2 Department of Nuclear Engineering, University of Tennessee, Knoxville, Tennessee 37996, United States; 3 Department of Physics and Astronomy, University of Tennessee, Knoxville, Tennessee 37996, United States; 4 Peter A. Rock Thermochemistry Laboratory and NEAT ORU, University of California Davis, Davis, CA 95616, United States

## Abstract

Fluorite-structured oxides find widespread use for applications spanning nuclear energy and waste containment, energy conversion, and sensing. In such applications the host tetravalent cation is often partially substituted by trivalent cations, with an associated formation of charge-compensating oxygen vacancies. The stability and properties of such materials are known to be influenced strongly by chemical ordering of the cations and vacancies, and the nature of such ordering and associated energetics are thus of considerable interest. Here we employ density-functional theory (DFT) calculations to study the structure and energetics of cation and oxygen-vacancy ordering in Ho_2_Zr_2_O_7_. In a recent neutron total scattering study, solid solutions in this system were reported to feature local chemical ordering based on the fluorite-derivative weberite structure. The calculations show a preferred chemical ordering qualitatively consistent with these findings, and yield values for the ordering energy of 9.5 kJ/mol-cation. Similar DFT calculations are applied to additional RE_2_Th_2_O_7_ fluorite compounds, spanning a range of values for the ratio of the tetravalent and trivalent (RE) cation radii. The results demonstrate that weberite-type order becomes destabilized with increasing values of this size ratio, consistent with an increasing energetic preference for the tetravalent cations to have higher oxygen coordination.

Fluorite-structured oxide compounds have been actively investigated for a range of technological applications, spanning solid-state ion conductors in electrochemical devices, to wasteforms and fuels for nuclear energy. In many such applications, the host tetravalent cations are substituted to varying degrees with trivalent cations such as rare earth (RE) atoms, which are charge-compensated by oxygen vacancies. Such substitutions can lead to the formation of configurationally long-range-ordered structures, such as pyrochlores^1^ or the *δ*-Zr_3_Y_4_O_12_ phase^2^, or disordered fluorite solid solutions that can be thermodynamically stable over extended composition ranges^3^. In the latter case, although configurational long-range order is absent on the cation and anion sublattices, experimental studies based on advanced structural characterization and thermochemistry techniques (e.g., refs 4, 5, 6, 7, 8 and references therein) have documented in several systems the presence of significant short-range order, i.e., local association and clustering of the different ionic species and oxygen vacancies. These experimental studies have been augmented by atomic-scale computational investigations (e.g., refs 9, 10, 11, 12, 13, 14, 15, 16 and references therein), which have established preferred structural motifs and associated energetic driving forces for defect association and compositional ordering across a broad range of chemistries.

The interest in chemical ordering in trivalent-substituted fluorite oxide solid solutions stems from its effects on the stability and physical properties of these materials. For example, short-range ordering significantly influences the overall energetic stability of yttria stabilized zirconia^4^, and it is expected to be similarly important to the phase stability of other related solid solutions more generally^8^. Further, the tendency for oxygen vacancies to bind preferentially to either the host or trivalent cation reduces the mobility of oxygen ions, which can be detrimental for performance in fuel cell electrolyte and oxygen sensor applications^17^, while it is believed to reduce oxidative corrosion rates in spent nuclear fuel^18^,^19^. Given the importance of these issues, detailed characterization of the nature, degree and spatial extent of short-range order in fluorite solid solutions is of considerable interest for the development of fluorite-structured oxides across a variety of applications.

In a very recent contribution, pair distribution function (PDF) analysis based on neutron total scattering measurements for the RE-substituted fluorite oxide system Ho_2_Zr_2_O_7_ has provided new insights into the nature of structural ordering^20^. In this study, the diffraction patterns for the samples studied displayed only peaks characteristic of the fluorite structure, indicative of the stability of a disordered fluorite solid-solution phase, consistent with previous reports (see, e.g., Stanek *et al*.^21^ and references therein). Nevertheless, the PDF analysis led to the conclusion that pronounced local structural ordering is present. Further, it was concluded that this local order can be described as being based on a weberite type fluorite-derivative superstructure. This discovery is particularly interesting in the context of previous investigations of the energetic stability of pyrochlore ordering in related systems with A_2_B_2_O_7_ compositions^1^, which have established that pyrochlore phases are stable when the ratio of the radius of the B^4+^ to A^3+^ cations is in the range of 0.56 to 0.68. For Ho_2_Zr_2_O_7_ the value of this ratio is significantly larger, at 0.83, and the observation of local short-range order of weberite type in this compound raises the question of whether this ordering may be found more generally in other RE-substituted systems with even larger tetravalent cations. Specifically, the possible relevance of weberite-type ordering for the structure of disordered fluorite phases observed in RE-substituted actinide-based fluorite solid solutions is of significant interest due to the potential importance for the stability and properties of nuclear fuel and wasteform materials.

In the present study we investigate these issues employing first-principles density-functional-theory (DFT) calculations. Specifically, we investigate the nature of the preferred fluorite-derivative structural ordering in Ho_2_Zr_2_O_7_, and calculate values for the energetic driving force underlying its formation, through the consideration of a set of hypothetical long-range-ordered configurations designed to probe energetically-preferred ordering motifs. We further show that the stability of the weberite-type ordering reported in ref. 20 is strongly influenced by the ratio of cation radii, by comparing the configurational energetics in Ho_2_Zr_2_O_7_ with those in RE-substituted ThO_2_ systems (RE=Ho, Y, Gd, Nd, and La) with the same stoichiometry. The focus on ThO_2_ host materials, which have received growing attention as fuel materials for nuclear energy production^18^,^19^, is motivated in part by the fact that this system provides access to larger ratios of the radii for the host tetravalent to the substituted RE trivalent cation. Further, although these systems are known to form disordered fluorite phases rather than ordered fluorite-derivative compounds under experimentally realized synthesis conditions (see, e.g., Aizenshtein *et al*.^22^ and references therein), previous calorimetric^22^ and computational^15^ studies have concluded that the thermodynamic stability of RE doped ThO_2_ solid solutions are influenced by short range ordering that varies with the size of the trivalent RE cation. A comparison of the DFT results presented here for both Ho_2_Zr_2_O_7_ and the RE_2_Th_2_O_7_ compounds shows a systematic trend for the weberite-type structural ordering reported in ref. 20 to be energetically destabilized with increasing ratio of the tetravalent to trivalent cation size.

## Results and Discussion

### Chemical ordering in Ho_2_Zr_2_O_7_

Motivated by the recent experimental observations reported by Shamblin *et al*.^20^, we begin by considering the energetics associated with the ordering of the Ho and Zr cations, and oxygen ions and vacancies, over the cation and anion sublattices of the fluorite structure for Ho_2_Zr_2_O_7_. Employing DFT calculations, as described in the Methods section, we compute the energetics for different cation and anion arrangements on the fluorite lattice, considering three different classes of configurations.

**Figure 1:**
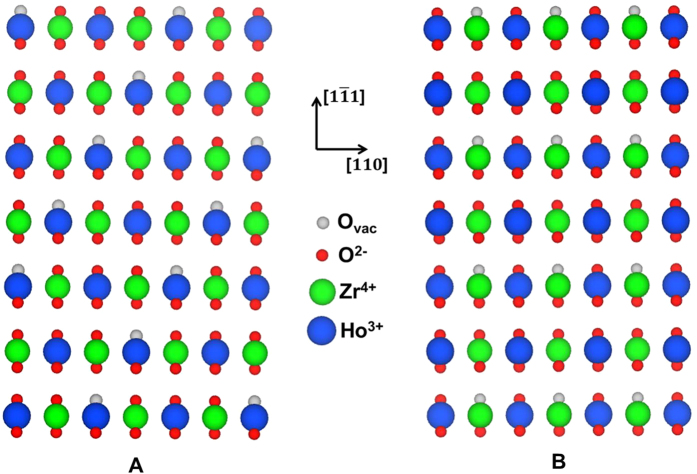
Projections of the lowest (**A**) and highest (**B**) energy A_2_B_2_O_7_-fluorite configurations for Ho_2_Zr_2_O_7_. Projections are shown along the 〈211〉 direction. For clarity, the ions are shown in their unrelaxed fluorite positions, and oxygen vacancies (denoted as O_*vac*_) are represented by gray circles.

The first set of configurations consists of all arrangements that can be generated as superstructures of the fluorite structure based on unit cells containing 2 tetravalent cations, 2 trivalent cations, 7 oxygen ions and 1 oxygen vacancy. Using a structural enumeration algorithm^23^ implemented in the Alloy Theoretic Automated Toolkit (ATAT)^24^,^25^, we find 27 possible configurations, which will be referred to in what follows as “A_2_B_2_O_7_-fluorite” configurations. We note that this set of enumerated configurations does not include the pyrochlore structure, which has a larger unit cell; pyrochlore ordering was not explicitly considered in this work as it is known to be energetically stable for B^4+^/A^3+^ size ratios smaller than those for Ho_2_Zr_2_O_7_ (see the Introduction section). We further note that although each of these is derived from different arrangements of Ho, Zr, oxygen and vacancies over the sublattices of the fluorite structure, the compositional ordering breaks the cubic symmetry such that the 27 configurations display a range of space group symmetries. As an example of the structures generated by this procedure we show in Fig. 1 two of the enumerated A_2_B_2_O_7_-fluorite configurations, which correspond to the lowest (A) and highest (B) energy structures obtained from DFT calculations for Ho_2_Zr_2_O_7_. Figure 1 illustrates that the structures correspond to different ordering patterns of the cations and oxygen vacancies. It is worth clarifying that the “A_2_B_2_O_7_-fluorite” structures should not be misinterpreted as disordered “defect fluorite”; rather, they represent ordered superstructures of fluorite.

**Figure 2:**
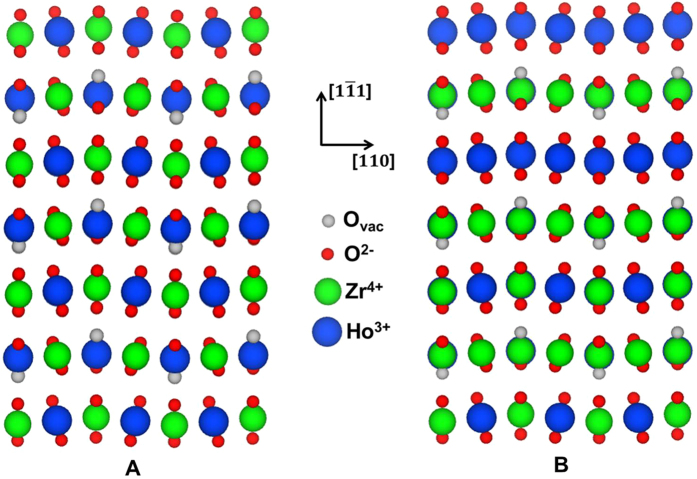
Projections of the lowest (**A**) and highest (**B**) energy weberite-type configurations for Ho_2_Zr_2_O_7_. Projections are shown along the 〈211〉 direction. For clarity, the ions are shown in their unrelaxed weberite positions, and oxygen vacancies (denoted as O_*vac*_) are represented by gray circles.

The second set of compositional arrangements considered are those associated with the weberite-type structure reported by Shamblin *et al*.^20^. Specifically, following ref. 20, we consider a conventional 44-atom unit cell based on the Ccmm space group, with Zr and Ho cations occupying the 4*a* and 4*b* sites, respectively, and sharing occupancy of the 8*g* sites. Oxygen ions fully occupy the 16*h* site, while three separate 4*c* sites are occupied by oxygen ions, and the fourth 4*c* site is vacant. The specific positions of the oxygen vacancies give rise to a structure in which the Ho atoms on the 4*b* sites are coordinated by 8 nearest-neighbor oxygen ions, the Zr atoms on the 4*a* site are coordinated by 6 oxygen ions, and the cations on the mixed 8*g* sites each have 7-fold coordination. In order to determine the most energetically stable weberite-type configuration based on this structure, we generate 70 (=*C*(8, 4)) configurations consisting of all possible arrangements of the four B^4+^ ions and four A^3+^ ions on the 8*g* sites, which we will refer to in what follows as “weberite-type” configurations. As an example of the structures generated by this procedure we show in Fig. 2 two of the weberite-type configurations, which correspond to the lowest (A) and highest (B) energy structures obtained from DFT calculations for Ho_2_Zr_2_O_7_. It should be noted that both the “weberite-type” and “A_2_B_2_O_7_-fluorite” configurations can be categorized as fluorite superstructures, and the distinction between them made here is purely for computational reasons, associated with the fact that they were generated by structural enumeration considering different constraints on the possible superstructure unit cells.

The third set of configurations considered in the calculations are so-called special-quasirandom structures (SQS)^26^. The SQS considered here are designed to be a periodic superstructure of fluorite with the atomic configuration of the B^4+^, A^3+^, oxygen and vacancy ions chosen to give short-ranged pair and multibody correlation functions matching as closely as possible those of a material with ideal, statistically random substitutional disorder on the cation and anion sublattices. The SQS structures are considered as they provide a means for computing from periodic-DFT calculations the energy of a phase with ideal random cation and anion distributions. The differences in energy between the SQS structures and the ordered configurations described above thus provide an estimate of the so-called “ordering energy,” which gives the energetic driving force for configurational ordering on the underlying parent (fluorite) structure. We consider two different SQS models taken from the literature^27^,^28^, one containing 297 atoms and the other 88 atoms. The former was shown by Wolff-Goodrich *et al*.^28^ to give more accurate estimates of the random phase energy, but it is significantly more computationally expensive than the latter. Therefore, the smaller 88-atom SQS is included because it will be used as the basis for exploring energetic trends in the next section.

**Figure 3:**
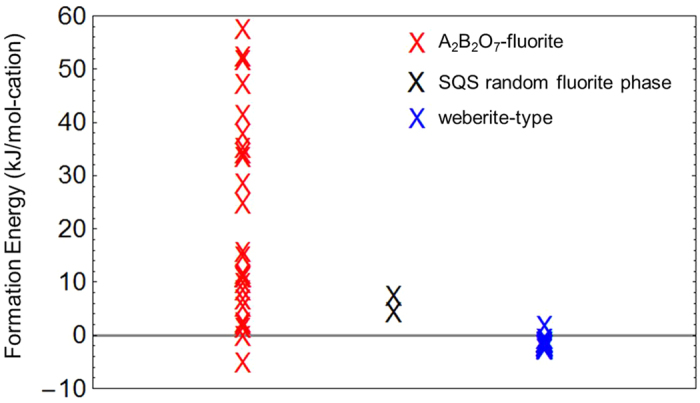
Formation energies (kJ/mol-cation) of all enumerated structures. A_2_B_2_O_7_-fluorite, weberite-type configurations, and the 88 atom (Δ*H*_*f*_ = 7.2 kJ/mol-cation) and 297 atom (Δ*H*_*f*_ = 4.0 kJ/mol-cation) SQS models of a random fluorite phase for Ho_2_Zr_2_O_7_ are shown.

The DFT calculated energies for all enumerated weberite-type, A_2_B_2_O_7_-fluorite, and SQS configurations for Ho_2_Zr_2_O_7_ are shown in Fig. 3. The results are presented as formation energies (Δ*H*_*f*_) with respect to constituent binary oxides, defined as follows for an A_2_B_2_O_7_ structure, with A and B denoting the trivalent and tetravalent cations, respectively:


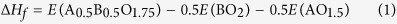


where the energies *E* and Δ*H*_*f*_ are defined per mole of cations. With this definition, positive values of Δ*H*_*f*_ indicate an energetic preference for phase separation into constituent binary oxides, while negative values indicate that the ordered configurations are stable with respect to phase separation and suggest an energetic tendency towards compound formation.

We start by considering the formation energy of the ideal random phase, which is predicted to be positive, with values of Δ*H*_*f*_ = 7.2 and 4.0 kJ/mol-cation for the 88 and 297 atom SQS models, respectively. Thus, the SQS models predict that an ideal random fluorite phase for Ho_2_Zr_2_O_7_ would have a positive enthalpy of formation, and would be unstable with respect to phase separation to the constituent binary oxides at low temperatures, but could be stabilized by its configurational entropy at high temperatures. Considering next the results for the different ordered configurations, it can be seen that the different weberite-type configurations are calculated to be very close in energy, which is not surprising since the nearest-neighbor oxygen coordination numbers around each cation are identical for each of the different structures (see above). By contrast, the spread in energy for the A_2_B_2_O_7_ fluorite configurations is much larger, as the structures generated in the enumeration approach include some atomic arrangements that are electrostatically very unfavorable. Importantly, it is found that the lowest energy A_2_B_2_O_7_-fluorite and weberite-type configurations have *negative* formation energies, in contrast to what was found for the ideal random phase (as modeled by the SQS structures). The results thus show that configurational ordering lowers the energy and leads to the energetic stabilization of Ho_2_Zr_2_O_7_ fluorite-derivative structures relative to phase separation to the constituent binary oxides. Taking the 297-atom SQS value as the best estimate for the formation energy of the ideal random solid phase, the calculations predict ordering energies of 9.5 and 6.7 kJ/mol-cation for the lowest energy A_2_B_2_O_7_-fluorite and weberite-type configurations, respectively.

The formation energies of the lowest-energy A_2_B_2_O_7_-fluorite and weberite-type configurations of Ho_2_Zr_2_O_7_ composition are shown in the top row of Table 1. It can be seen that Δ*H*_*f*_ for the lowest-energy A_2_B_2_O_7_-fluorite configuration is slightly lower (i.e., the structure is energetically more stable) than that found for the lowest-energy weberite-type configuration, by approximately 3 kJ/mol-cation. This DFT result may seem at odds with the conclusion of Shamblin *et al*.^20^ that the local order in the Ho_2_Zr_2_O_7_ is of weberite type, since we are finding another configuration with lower energy. However, despite their different space-group symmetries, the configurational arrangement of the atoms for the lowest-energy A_2_B_2_O_7_-fluorite configuration is in fact very similar to what is found for the lowest-energy weberite-type configuration. It was found through simulations of the PDFs that both the A_2_B_2_O_7_-fluorite and weberite-type configurations lead to a comparable level of agreement with the experimental data reported in ref. 20, such that the difference in ordering is expected to be too subtle to distinguish from the neutron total scattering analysis.

The similarity of the lowest-energy A_2_B_2_O_7_-fluorite and weberite-type configurations can be appreciated by comparing their projected images shown in Figs 1(A) and 2(A), respectively. Both are seen to show identical “checkerboard” configurations for the Ho and Zr cations in this projection, and display exactly the same ordering of the cations over the sites of the fluorite lattice. Note that the lowest-energy A_2_B_2_O_7_-fluorite configuration is relaxed and therefore more distorted from what is shown in the figure, tending towards the lower-symmetry, weberite-type ordering as well. The primary difference between the two structures is seen to be associated with the ordering of the oxygen vacancies. Specifically, the oxygen vacancies in the lowest-energy A_2_B_2_O_7_-fluorite configuration are ordered as second nearest neighbors along the 〈110〉 direction in the lowest-energy A_2_B_2_O_7_-fluorite configuration, whereas for the lowest-energy weberite-type configuration they are ordered as third nearest neighbors along 〈111〉. This difference in vacancy ordering is relatively subtle, as the number of nearest-neighbor oxygen ions around Zr is identical in both structures. Specifically, both structures feature the same preference for Zr (Ho) to be surrounded by less (more) oxygen ions in its nearest-neighbor shell than the average concentration. In both the lowest-energy A_2_B_2_O_7_-fluorite and weberite-type structures the average number of oxygen ions surrounding Zr (Ho) is 6.5 (7.5), compared to the number of 7 that would characterize a random distribution. This preference for lower (higher) coordination around the B^4+^ (A^3+^) cation is consistent with reverse Monte Carlo (RMC) analysis from neutron total scattering^7^ and X-ray absorption experiments^29^,^30^ on zirconate and hafnate systems (i.e., A_2_B_2_O_7_, B=Zr, Hf), with a general trend towards increasing (decreasing) oxygen ion coordination of B (A) as the A cation size decreases (this trend is discussed in further detail in the next section). Similar coordination behavior was also found for titanate systems^31^; however, the local order was found to be pyrochlore, which is distinctly different compared to the preferred weberite-type configuration found here and in ref. 29 for Ho_2_Zr_2_O_7_ (a comparison of the local order for pyrochlore and weberite-type configurations is included in the next section).

### Chemical ordering and relative stability of weberite-type structures in RE_2_Th_2_O_7_

**Figure 4:**
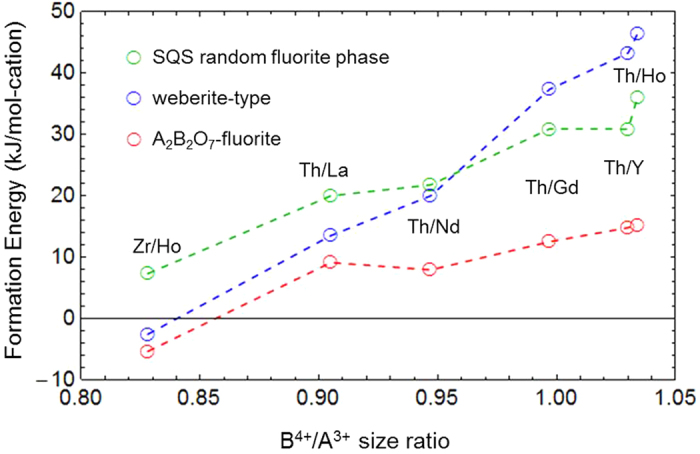
Formation energy vs. cation size ratio. Formation energies (kJ/mol-cation) of lowest-energy A_2_B_2_O_7_-fluorite, lowest-energy weberite-type, and SQS random phase configurations (88 atom) as a function of B^4+^/A^3+^ size ratio are plotted for Ho_2_Zr_2_O_7_ and RE_2_Th_2_O_7_ systems.

In this section we consider the results obtained by applying the computational approach described above to the study of the energetics of chemical ordering in RE_2_Th_2_O_7_ systems, where the trivalent RE cations considered are listed along with their sizes relative to tetravalent Zr and Th in Table 2. We chose thorium as a representative actinide element because it has only one oxidation state (4+) and no *f*-electrons to complicate the DFT calculations. For each choice of RE trivalent cation we performed DFT calculations to identify the lowest-energy A_2_B_2_O_7_-fluorite and weberite-type configurations from amongst the enumerated structures described in the previous section. The formation energies for these lowest-energy structures are listed in Table 1, and are plotted in Fig. 4, along with the corresponding values for the 88-atom SQS structure (approximating a random fluorite phase), as a function of the ratio of the tetravalent to trivalent cation ionic radius (referred to hereafter at the B^4+^/A^3+^ size ratio). The results for Ho_2_Zr_2_O_7_ are also included in Fig. 4 for comparison.

Figure 4 displays clear trends with increasing values of the B^4+^/A^3+^ size ratio. First, all of the Δ*H*_*f*_ values calculated in the RE_2_Th_2_O_7_ systems are positive, with a magnitude that tends to increase with increasing value of the B^4+^/A^3+^ size ratio. In other words, the fluorite-derivative structures considered here for RE_2_Th_2_O_7_ are found to become increasingly unstable, with respect to phase separation to constituent binary oxides, as the size of the trivalent RE cation decreases. This trend is consistent with the analysis of previous DFT and calorimetric studies on similar defected fluorite oxide systems given by Zhang *et al*.^32^. The general trend can be rationalized based on simple ionic coordination arguments. Specifically, tetravalent (trivalent) cations tend to reduce (increase) the nearest-neighbor oxygen ion coordination relative to the constituent binary oxides upon mixing, and therefore systems with smaller (larger) B^4+^ (A^3+^) cations are expected to be more energetically stable. The net effect is that, amongst the systems considered in this work, only for Ho_2_Zr_2_O_7_, where the B^4+^/A^3+^ size ratio has the minimum value of 0.828, do the fluorite-derivative structures have negative formation energies and are thus energetically stable with respect to phase separation to constituent binary oxides. A negative formation energy for a similar system, Y_2_Zr_2_O_7_, was calculated previously with a magnitude similar to Ho_2_Zr_2_O_7_^11^,^12^,^13^. The effect of changing Zr to the larger Th cation is to destabilize these structures, to a degree that increases with decreasing size of the RE ion.

The second trend observed in Fig. 4 is the increasing difference between Δ*H*_*f*_ for the lowest-energy weberite-type and A_2_B_2_O_7_-fluorite configurations with increasing B^4+^/A^3+^ size ratio. While the energies for these two configurations are close for Ho_2_Zr_2_O_7_, the weberite-type configurations become increasingly higher in energy than the lowest-energy A_2_B_2_O_7_-fluorite configuration as the size of the RE ion decreases in the RE_2_Th_2_O_7_ systems. The same trend is also apparent when comparing the energies of the weberite-type configurations with the SQS model of the ideal random phase: while the weberite-type configurations are lower in energy than the random phase for smaller values of the B^4+^/A^3+^ size ratio, they become higher in energy for larger size ratios. These overall trends can be understood based on a consideration of the oxygen neighbor coordinations around the different cation species in the different types of configurations, which is described next.

The weberite-type configurations all feature an average number of 7.5 oxygen neighbors surrounding the trivalent cation, and 6.5 oxygen neighbors surrounding the tetravalent cation (see the description of the weberite-type structure given in the previous section). By contrast, the lowest-energy A_2_B_2_O_7_-fluorite configuration for RE_2_Th_2_O_7_ (which is the same configuration for all of the RE elements considered) features a larger oxygen coordination surrounding tetravalent Th than the trivalent RE cations, namely 7.5 (6.5) oxygen neighbors surrounding the tetravalent (trivalent) cation. Recall that the random phase has the average number of 7 oxygen ions around both trivalent and tetravalent cations. Consistent with the coordination arguments summarized above, the DFT results suggest that while the larger oxygen coordination around the trivalent RE cation is favored energetically for the Ho_2_Zr_2_O_7_ system, it is disfavored in the RE_2_Th_2_O_7_ systems, due to the larger size of the Th cation relative to Zr, to a degree that increases with decreasing size of the RE ion.

To put the energy trends related to oxygen coordination into a broader context, we summarize the nature of the preferred compositional ordering in the current and related systems in Table 3. In this table, we report for the weberite-type structure, and lowest-energy A_2_B_2_O_7_-fluorite type structures found here the preferred oxygen coordination number, as described through a short-range-order (SRO) parameter *α*_*B*_. By analogy with the Warren-Cowley^33^ parameter commonly used to describe SRO in alloys, we define *α*_*B*_ as follows:


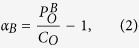


where 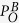 is the fraction of nearest-neighbor anion-sublattice sites surrounding a tetravalent B cation (Zr or Th in the current study) that are occupied by oxygen ions, while *C*_*O*_ is the average concentration (site fraction) of anion sublattice sites occupied by oxygen. For an A_2_B_2_O_7_ fluorite phase with ideal random substitutional disorder, *α*_*B*_ is identically zero, and positive (negative) values indicate a preferential coordination of oxygen around tetravalent B (trivalent A) cations.

The results in Table 3 show positive and negative values for the lowest-energy A_2_B_2_O_7_-fluorite configurations for RE_2_Th_2_O_7_ and Ho_2_Zr_2_O_7_, respectively, and negative values are associated with weberite-type configurations. Also included in Table 3 are values of *α*_*B*_ for pyrochlore and *δ*-Zr_3_Y_4_O_12_ compounds, which are additional experimentally-observed fluorite-derivative structures featuring higher oxygen coordination around trivalent relative to tetravalent cations. It is well documented in the literature that pyrochlore systems are stable for B^4+^/A^3+^ size ratios smaller than those characteristic of the systems considered here^1^ (c.f., the discussion in the Introduction section). It is perhaps not surprising that the *δ*-Zr_3_Y_4_O_12_ phase and lowest-energy A_2_B_2_O_7_ configuration for Ho_2_Zr_2_O_7_ have a similar oxygen coordination preference (i.e., reduced (increased) oxygen coordination for the tetravalent (trivalent) cation relative to the random phase), given that the Ho and Y cations have similar ionic radii (1.015 and 1.019 Å, respectively^34^). Given this observation, and previous work by Stanek *et al*.^21^ predicting stability of *δ*-phase structures for systems with cation size ratios bordering on those considered here, a more complete understanding of ordering tendencies in these systems would benefit from future work investigating the competition between *δ*-phase related ordering and the structures studied here for A_2_B_2_O_7_ compositions. Overall, the results in Fig. 4 and Table 3 illustrate the trend that as the B^4+^/A^3+^ size ratio increases from the small values characteristic of pyrochlore ordering to the much larger values characterizing the RE_2_Th_2_O_7_ systems discussed in this section, there is an increasing preference for lowering the coordination of oxygen ions around the trivalent cation in favor of increasing the coordination around the tetravalent cation.

## Summary and Conclusions

Density-functional-theory calculations have been undertaken to investigate the ordering energetics of tetravalent and trivalent cations and oxygen vacancies on fluorite-structured Ho_2_Zr_2_O_7_ and RE_2_Th_2_O_7_ (with RE=Ho, Y, Gd, Nd, and La). In these systems, formation energies were computed for a set of ordered A_2_B_2_O_7_-fluorite derivative configurations, representing all possible arrangements of the tetravalent (B) and trivalent (A) cations and oxygen vacancies over the sites of the fluorite structure with 11-atom primitive unit cells, as well as a set of configurations enumerated by considering all possible cation arrangements over the mixing site of the weberite structure reported by Shamblin *et al*.^20^. For Ho_2_Zr_2_O_7_, the results establish an energetic driving force for ordering on the fluorite lattice that is characterized by cation arrangements illustrated in Fig. 1(A), and a preference for a higher coordination of Ho by oxygen relative to Zr cations. The associated magnitude of the ordering energy is calculated to be approximately 9.5 kJ/mol-cation. The results are qualitatively consistent with the findings reported in the work of Shamblin *et al*.^20^, in that the lowest-energy configuration obtained from the enumerated ordered fluorite superstructures of A_2_B_2_O_7_ features the same type of cation ordering and the same average number of nearest-neighbor oxygen ions surrounding Ho and Zr as the lowest energy weberite configuration identified in the DFT calculations.

For the RE_2_Th_2_O_7_ systems, the DFT calculations show that as the value of the B^4+^/A^3+^ size ratio increases, there is a growing energetic preference for the tetravalent cation to have higher oxygen coordination relative to the trivalent cation. This leads to a destabilization of weberite-type order relative to ideally random phases and ordered configurations with higher average oxygen coordination around Th cations. A comparison of the lowest-energy configurations obtained here with those found in the long-range-ordered *δ*-Zr_3_Y_4_O_12_ and pyrochlore phases, which are observed to be stable in systems with B^4+^/A^3+^ size ratios comparable to or smaller than Ho_2_Zr_2_O_7_ compounds, shows a more general trend for the increasing preference for higher oxygen coordination around the tetravalent (trivalent) cation as the size of B^4+^ increases (decreases) relative to A^3+^. This general trend is consistent with previous experimental investigations on RE_2_Hf_2_O_7_ and RE_2_Zr_2_O_7_^29^,^30^, which show that increasing size of the RE cation increases the preferred coordination around it. Overall, the results of the current study suggest that the weberite-type order reported by Shamblin *et al*.^20^ in Ho_2_Zr_2_O_7_ is expected to be energetically preferred in trivalent-substituted fluorite-oxide phases that have B^4+^/A^3+^ size ratios intermediate between the smaller values characteristic of pyrochlore ordering and the larger values associated with the RE_2_Th_2_O_7_ systems considered here.

## Methods

The computational approach employed in this work is designed to understand trends in the ordering of the tetravalent and trivalent cations and oxygen vacancies across the range of chemistries for the A_2_B_2_O_7_ systems considered. These trends in preferred ordering motifs are investigated through the consideration of a set of hypothetical long-range-ordered fluorite superstructures, designed to probe different coordinations of oxygen and vacancies around the trivalent and tetravalent cations, as well as different cation ordering patterns. The energetic driving forces associated with these different ordering motifs are investigated by comparing the energies of the hypothetical long-range-ordered phases with a random solid-solution phase modeled with SQS structures, as described in the main text. We note that this approach cannot be used to fully describe the state of short-range order present in the experimental samples, including in particular the spatial range of the short-range-ordered domains. Rather, the goal is to understand the energetics underlying the formation of such short-range order, as well as the energetically preferred anion and cation ordering configurations. In this approach, the formation energies of relaxed structures were obtained using DFT within the formalism of the projector augmented-wave (PAW) method^35^,^36^ and the Perdew-Burke-Ernzerhof (PBE) generalized gradient approximation (GGA)^37^,^38^, as implemented in the Vienna *ab initio* simulation package (VASP)^39^,^40^. The PAW potentials use 4 valence electrons for Zr (5s^2^4d^2^), 12 for Th (6s^2^6p^6^6d^2^7s^2^), 11 for Y (4s^2^4p^6^5s^2^4d^1^) and La (5s^2^5p^6^6s^2^5d^1^) and 6 for O (2s^2^2p^4^). For Ho, Gd, and Nd, the occupied 4*f* orbitals in the trivalent oxidation state are treated as core electrons. We employ a plane-wave cutoff energy of 400 eV and 500 eV for Ho_2_Zr_2_O_7_ and RE_2_Th_2_O_7_, respectively, and the Brillouin zone is sampled using the Monkhorst-Pack scheme with relatively coarse k-point meshes of at least 4 × 4 × 4 and 1 × 2 × 2 for the A_2_B_2_O_7_-fluorite and weberite-type structures, respectively, to initially screen out energetically disfavorable structures. The lowest energy Ho_2_Zr_2_O_7_ structures were then sampled with k-point meshes of 8 × 8 × 8 and 4 × 4 × 4 for the A_2_B_2_O_7_-fluorite and weberite-type structures, respectively. In the RE_2_Th_2_O_7_ systems, k-points meshes of 4 × 4 × 4 were found to be sufficient to achieve well converged energies. The k-point meshes used for the end members for the formation energy calculations are 8 × 8 × 8 for cubic ZrO_2_, 6 × 6 × 6 for cubic ThO_2_, 4 × 4 × 4 for C-type Ho_2_O_3_, Y_2_O_3_, and Gd_2_O_3_, and 8 × 8 × 8 for A-type Nd_2_O_3_ and La_2_O_3_. From convergence checks with respect to plane-wave cutoff and k-point sampling, the energy differences between low energy structures are estimated to be converged to within 0.3 kJ/mol-cation. Electronic self-consistency was considered achieved when the total energy change between electronic steps is within 10^−4^ eV, and relaxation of ionic positions and cell shape were carried out with no symmetry constraints until residual forces were below approximately 15 meV/Å.

## Additional Information

**How to cite this article:** Solomon, J. M. *et al*. Chemical ordering in substituted fluorite oxides: a computational investigation of Ho_2_Zr_2_O_7_ and RE_2_Th_2_O_7_ (RE=Ho, Y, Gd, Nd, La). *Sci. Rep.* **6**, 38772; doi: 10.1038/srep38772 (2016).

**Publisher's note:** Springer Nature remains neutral with regard to jurisdictional claims in published maps and institutional affiliations.

## Figures and Tables

**Table 1:** Formation energies (kJ/mol-cation) for the lowest-energy fully relaxed A_2_B_2_O_7_-fluorite and weberite-type configurations.

B^4+^	A^3+^	Δ*H*_*f*_ (kJ/mol-cation) A_2_B_2_ O_7_-fluorite	Δ*H*_*f*_ (kJ/mol-cation) weberite	Δ*H*_*f*_ (kJ/mol-cation) Difference	B^4+^/A^3+^ Size Ratio
Zr	Ho	−5.4	−2.6	2.8	0.828
Th	La	9.1	13.5	4.4	0.905
Th	Nd	7.9	20.0	12.1	0.947
Th	Gd	12.5	37.4	24.9	0.997
Th	Y	14.8	43.3	28.5	1.030
Th	Ho	15.2	46.5	31.3	1.034

“Δ*H*_*f*_ difference” denotes the difference between the A_2_B_2_O_7_-fluorite and weberite-type formation energies (“Δ*H*_*f*_ A_2_B_2_O_7_-fluorite” and “Δ*H*_*f*_ weberite”).

**Table 2:** Charges and sizes of cations considered in this work.

	Zr	Th	Ho	Y	Gd	Nd	La
Charge	4+	4+	3+	3+	3+	3+	3+
Size (Å)	0.84	1.05	1.015	1.019	1.053	1.109	1.16

The sizes represent Shannon eightfold-coordination ionic radii^34^.

**Table 3:** Short-range-order parameters *α*_*B*_ of the first nearest-neighbor anion shell.

Structure	*α*_*B*_
Pyrochlore	−0.14
weberite-type configuration	−0.07
Lowest-energy A_2_B_2_O_7_ configuration for Ho_2_Zr_2_O_7_	−0.07
*δ*-Zr_3_Y_4_O_12_	−0.03
Ideal random fluorite phase	0
Lowest-energy A_2_B_2_O_7_ configuration for RE_2_Th_2_O_7_	0.07

*α*_*B*_ is calculated based on the anion shell around tetravalent cations (B^4+^) in pyrochlore, weberite-type, *δ*-Zr_3_Y_4_O_12_, ideal random phase, and computed lowest-energy configurations for the A_2_B_2_O_7_ systems considered in this work.
