# Corrigendum: Chemical ordering in substituted fluorite oxides: a computational investigation of Ho_2_Zr_2_O_7_ and RE_2_Th_2_O_7_ (RE = Ho, Y, Gd, Nd, La)

**DOI:** 10.1038/srep42380

**Published:** 2017-02-13

**Authors:** Jonathan M. Solomon, Jacob Shamblin, Maik Lang, Alexandra Navrotsky, Mark Asta

Scientific Reports 6: Article number: 3877210.1038/srep38772; published online: 12 12 2016; updated: 02 13 2017

This Article contains errors in Reference 31 which was incorrectly given as:

‘Sanjuán, M. L. *et al*. Raman and x-ray absorption spectroscopy study of the phase evolution induced by mechanical milling and thermal treatments in R2Ti2O7 pyrochlores. *American Physical Society* **84**, 104207 (2013)’.

The correct reference is listed below:

Sanjuán, M. L. *et al*. Raman and x-ray absorption spectroscopy study of the phase evolution induced by mechanical milling and thermal treatments in R2Ti2O7 pyrochlores. *Phys. Rev.* B **84**, 104207 (2011).

